# Maternal experiences and family dynamics following Down syndrome diagnosis in Saudi Arabia

**DOI:** 10.3389/fpsyg.2026.1752134

**Published:** 2026-04-10

**Authors:** Nuha Alrayes, Reem Alyoubi, Anas Alyazidi, Bassam Jamalalail, Husna Irfan Thalib, Noor Ahmad Shaik, Wisam Habhab, Ashwaq Alsabban, Yaser Alkhiary, Noha M. Issa

**Affiliations:** 1 Department of Medical Laboratory Sciences, Faculty of Applied Medical Sciences, King Abdulaziz University, Jeddah, Saudi Arabia; 2 Princess Al-Jawhara Center of Excllence in Research of Hereditary Disorders, King Abdulaziz University, Jeddah, Saudi Arabia; 3 Institute of Genomic Medicine Sciences, King Abdualaziz University, Jeddah, Saudi Arabia; 4 Department of Pediatric, Faculty of Medicine, King Abdulaziz University, Jeddah, Saudi Arabia; 5 Faculty of Medicine, King Abdulaziz University, Jeddah, Saudi Arabia; 6 Center for Applied and Translational Genomics, Mohammed Bin Rashid University of Medicine and Health Sciences, Dubai, United Arab Emirates; 7 General Medicine Practice Program, Batterjee Medical College, Jeddah, Saudi Arabia; 8 Department of Genetic Medicine, Faculty of Medicine, King Abdulaziz University, Jeddah, Saudi Arabia; 9 Department of Biological Science, Faculty of Sciences, King Abdulaziz University, Jeddah, Saudi Arabia; 10 Oral and Maxillofacial Prosthodontics Department, Faculty of Dentistry, King Abdulaziz University, Jeddah, Saudi Arabia; 11 Department of Genetic Medicine, King Abdulaziz University Hospital, Jeddah, Saudi Arabia

**Keywords:** caregiver, Down syndrome, emotional impact, intellectual disability, mothers, trisomy 21

## Abstract

**Background:**

Down syndrome (DS) is a genetic condition characterized by developmental delays and congenital irregularities. Parents of children with DS face significant psychological challenges, with the method of diagnosis delivery critically influencing their initial reactions and long-term coping strategies.

**Objective:**

This study aimed to investigate the emotional impact on mothers following a DS diagnosis in their child.

**Methods:**

A cross-sectional study was conducted with 161 mothers of children with DS in Saudi Arabia. Participants completed a survey assessing demographic characteristics, emotional experiences upon diagnosis, and the impact on family and social life. Data were analyzed using descriptive statistics and Pearson’s chi-square tests.

**Results:**

Most participants were Saudi nationals (80.5%). Most mothers were aged 30–40 years at childbirth, while their husbands were typically 40–50 years old. A substantial proportion of mothers (41.6%) reported marital tension, with 58.3% of these experiencing a weakened partner bond. Lack of emotional support from partners was reported by 63.3% of participants. Key correlations revealed that detailed diagnostic explanations were associated with reduced maternal self-blame (*p* = 0.007), while maternal acceptance correlated with decreased persistent anxiety (*p* = 0.0001).

**Conclusion:**

The findings highlight the critical importance of compassionate, clear diagnosis delivery and comprehensive support for mothers of children with DS. Healthcare providers should prioritize empathetic communication and provide adequate resources, while also encouraging mutual support between partners to mitigate stress and promote child acceptance. These measures are essential for improving family outcomes following a DS diagnosis.

## Introduction

Down syndrome (DS) is a genetic condition resulting from trisomy 21, characterized by developmental delays, intellectual disability, and various congenital anomalies. With an estimated global incidence of approximately 1 in 700 to 1 in 1000 live births, it represents the most common chromosomal cause of intellectual disability worldwide. While the medical and developmental aspects of DS have been extensively studied, less attention has been paid to the experiences of families receiving this diagnosis, particularly in non-Western contexts.

The experience of mothers of children with DS in Saudi Arabia may be uniquely shaped by cultural, socioeconomic, and healthcare factors that distinguish it from patterns documented in the largely Western-centric literature. A diagnosis of DS is a life-altering event associated with significant psychological strain on parents (Fortier and Wanlass, 1984; Mulcahy and Savage, 2016), though how this strain manifests and is managed varies considerably across contexts. We focus specifically on mothers rather than both parents for several reasons. In Saudi Arabia, mothers typically serve as primary caregivers and are often more directly involved in the daily care of children with special needs. Additionally, previous research suggests that mothers and fathers may experience and cope with a child’s disability differently (Almalki, 2020) and understanding maternal experiences in particular can inform targeted support interventions. The wide maternal age range in our sample (spanning from <20 to 50 years) reflects the natural demographic diversity of childbearing in Saudi society and allows for examination of how maternal experiences may vary across different life stages and family circumstances. Finally, practical considerations regarding access to participants through mother-focused support networks made this focus feasible.

Cultural context is particularly relevant. While international studies document common maternal responses such as shock, denial, and anxiety following diagnosis, often exacerbated by insensitive communication from healthcare professionals (Huiracocha et al., 2017; Skotko, 2005), these findings typically reflect individualistic coping frameworks where stress is viewed as a personal burden. In contrast, Saudi society is characterized by strong extended family networks and communal approaches to child-rearing (Alqarawi et al., 2023), and religious beliefs emphasizing patience and acceptance have been suggested as potential influences on coping in Middle Eastern populations (Al-Gamal and Long, 2013). Whether these cultural factors buffer or otherwise modify maternal experiences remains an empirical question.

Socioeconomic resources also shape adaptation. Raising a child with a disability entails emotional, physical, and financial demands (Cuskelly et al., 2009), and access to specialized care, therapy, and educational support in Saudi Arabia is often tied to a family’s socioeconomic position (Al-Jadid and Robert, 2010). A partner’s educational level may serve as a particularly relevant marker in this context, potentially reflecting greater awareness of developmental needs and access to resources that support maternal coping.

The quality of healthcare encounters further influences outcomes. International research indicates that how a diagnosis is shared substantially affects families’ initial responses and can contribute to long-term resentment when handled poorly (Huiracocha et al., 2017; Baird et al., 2000). However, the specific protocols, training practices, and support services available in Saudi Arabia have received limited empirical attention. The effectiveness of prenatal screening, the preparation of healthcare professionals in delivering sensitive diagnoses, and the availability of ongoing support networks all warrant investigation within this specific healthcare context. Additionally, given the cultural significance of gender in Saudi society, child sex was included as a variable of interest, as prior research suggests it may influence parental responses (Almalki, 2020).

To provide a theoretical framework for understanding these dynamics, we draw upon Lazarus and Folkman’s transactional model of stress and coping (Lazarus and Folkman, 1984). This model posits that when individuals encounter a stressful event (such as a DS diagnosis), they engage in primary appraisal (evaluating the significance of the event), secondary appraisal (assessing available resources to cope), and subsequently employ coping strategies that shape psychological outcomes. Within this framework, cultural factors (such as extended family support and religious beliefs), socioeconomic resources (including partner education), and healthcare experiences (the quality of diagnosis disclosure) can be understood as influencing both appraisal processes and available coping resources. The model thus provides a coherent lens through which to examine how these various factors collectively shape maternal adjustment following a DS diagnosis. This framework has been widely applied in studies of parental adaptation to childhood disability, demonstrating its utility in understanding how families mobilize resources in response to diagnosis-related stress (lden, 1997; McCubbin et al., 1997).

Given the exploratory nature of this research in an understudied population, we did not formulate specific *a priori* hypotheses. Rather, this study aimed to examine the emotional impact on mothers following a DS diagnosis in Saudi Arabia and to explore associations with key demographic and contextual variables. By exploring how mothers’ experiences intersect with healthcare delivery systems, educational support structures, and broader societal attitudes toward disability, we seek to illuminate the multi-level factors that shape family adaptation. Specifically, we investigated: (1) the circumstances and perceived quality of diagnosis disclosure; (2) maternal emotional responses, both initially and ongoing; (3) the role of partner support and family dynamics; and (4) associations with key demographic variables, including partner’s educational level, maternal employment status, and child sex. By focusing on this understudied population, we sought to generate evidence that could inform culturally sensitive clinical practices and family support interventions.

## Materials and methods

### Study design and setting

This study employed a cross-sectional design based on data collected via an online survey distributed in Saudi Arabia. Ethical approval was obtained from the Unit of Biomedical Ethics Research Committee in the Faculty of Medicine at King Abdulaziz University, and the study adhered to the Strengthening the Reporting of Observational Studies in Epidemiology (STROBE) reporting guideline for cross-sectional studies (Von Elm et al., 2007). All participants provided electronic informed consent before commencing the survey.

### Study population and procedure

The target population was mothers of children diagnosed with DS residing in Saudi Arabia. A total of 161 mothers completed the survey. As detailed in Table 1, participants were predominantly Saudi nationals (80.5%), with most mothers aged 30–40 years at the time of the child’s birth (47.5%) and holding a bachelor’s degree (52.2%). Approximately one-third of participants (33.5%) were employed outside the home.

**Table 1:** Sociodemographic and clinical characteristics of the study participants.

Variable	Group	Frequency (*n*)	Percent (%)
Child’s characteristics			
Age (years)	1–5	94	58.4
	5–10	28	17.4
	10–15	24	14.9
	≥ 20	15	9.3
Sex	Male	99	61.5
	Female	62	38.5
Nationality	Saudi	128	80.5
	Non-Saudi	31	19.5
Another child with DS	No	154	95.7
	Yes	7	4.3
Maternal characteristics			
Age (years)	<20	2	1.3
	20–30	29	18.1
	30–40	76	47.5
	40–50	53	33.1
Education level	Uneducated	4	2.5
	Below HIGH SCHOOL	18	11.2
	High school	33	20.5
	Bachelor’s degree	84	52.2
	Master’s degree	13	8.1
	Higher degree	9	5.6
Employment status	Not working	107	66.5
	Working	54	33.5
Paternal characteristics			
Age (years)	20–30	14	8.7
	30–40	46	28.6
	40–50	80	49.7
	50–60	18	11.2
	60–70	3	1.9
Education level	Uneducated	1	0.6
	Below high school	19	11.8
	High school	33	20.5
	Bachelor’s degree	72	44.7
	Master’s degree	20	12.4
	Higher degree	16	9.9
Family characteristic			
Consanguinity	No	113	70.2
	Yes	48	29.8

An online questionnaire was developed using Google Forms and distributed through multiple channels, including DS-specific parent support groups on social media platforms (primarily WhatsApp and Telegram groups dedicated to parents of children with disabilities), patient advocacy organizations, and hospital-based pediatric and genetic clinics in major cities across Saudi Arabia. The survey link remained active between January and October 2025. Participation was entirely voluntary and anonymous, and no incentives were offered to participants. Based on pilot testing with 15 mothers, the survey took approximately 9 min to complete.

### Questionnaire development

The questionnaire was developed specifically for this study through a multi-stage process designed to ensure content validity and cultural appropriateness. An initial pool of items was generated based on an extensive review of the literature examining parental experiences following DS diagnosis, with particular attention to studies conducted in Middle Eastern contexts to capture relevant cultural dimensions. The domains assessed included demographic and clinical characteristics, circumstances and quality of diagnosis disclosure, initial and ongoing maternal emotional responses, partner support and family dynamics, and impact on social and professional life.

The initial draft was subsequently reviewed by a panel of five experts comprising two geneticists, one clinical psychologist, one public health specialist, and one social worker with expertise in family support. These panel members evaluated each item for clarity, relevance, and cultural sensitivity, and their feedback was incorporated to refine the instrument. The revised questionnaire was then pilot tested with 15 mothers of children with DS who were not included in the final study sample. These pilot participants completed the survey and were interviewed to identify any ambiguous wording, technical difficulties, or excessive survey burden. Minor modifications to phrasing and response options were made based on this feedback, and pilot data were not included in the final analysis.

No previously validated instruments were incorporated into the questionnaire, as the study aimed to capture culturally specific experiences not adequately addressed by existing Western-developed measures. Instead, the questionnaire consisted of purpose-built items with response formats including dichotomous (yes/no), multiple-response (select all that apply), and three-point Likert-type scales (agree/neutral/disagree). The specific items and their response options are detailed below by section.

Section A: Demographic and clinical characteristics (16 items) collected information on: maternal age (categorical: <20, 20–30, 30–40, 40–50 years); maternal education (uneducated, below high school, high school, bachelor’s degree, master’s degree, higher degree); maternal employment status (not working/working); paternal age and education (same categories); child’s age (1–5, 5–10, 10–15, ≥20 years); child’s sex (male/female); nationality (Saudi/non-Saudi); presence of other children with DS (yes/no); consanguinity (yes/no); and timing of diagnosis (before birth, at birth, 24 h after birth, 48 h after birth, 1 month after birth, more than 1 year after birth).

Section B: Diagnosis experience and initial emotional impact (12 items) assessed: the professional who delivered the diagnosis (pediatrician, gynecologist/obstetrician, general physician/nurse, family/friends); perceived manner of delivery (good/merciful way, uncaring/unsympathetic way, horrible way); whether information or resources were provided (excellent explanation about DS, no explanation or resources provided); and initial emotional reactions (multiple response options including sadness, shock, did not know what DS was, acceptance, depression).

Section C: Ongoing impact on family and social life (18 items) evaluated: current maternal emotional well-being (items on anxiety, guilt, anger, self-blame with yes/no response); partner support and reactions (multiple response options including acceptance, calmed mother down, denial, depression); effects on marital relationship (yes/no; if yes, whether relationship strengthened or weakened); changes in social relationships (with partner’s family, mother’s own family, friends); and for employed mothers, impact on professional life (whether work life affected, and if so, nature of impact including losing focus, decreasing hours, quitting job).

### Psychometric considerations

It is important to acknowledge that formal psychometric validation, such as factor analysis or assessment of internal consistency using Cronbach’s alpha, was not performed on this newly developed instrument. The questionnaire was designed as a descriptive and exploratory tool intended to capture the specific experiences of this understudied population within its unique cultural context. Many items were intended to be analyzed individually rather than combined into multi-item scales, which somewhat limits the applicability of traditional reliability measures. Consequently, the findings presented here should be interpreted as exploratory in nature, and associations between variables should be viewed with appropriate caution. Future research in this area would benefit from employing validated instruments for key constructs such as anxiety, depression, and caregiver burden to enable more robust comparisons across different populations and settings.

### Statistical analysis

Before analysis, data were exported from Google Forms to Statistical Package for the Social Sciences (SPSS) version 26 and checked for completeness and accuracy. No missing data were present as all survey items were mandatory, resulting in a complete dataset for all 161 participants. Descriptive statistics, including frequencies and percentages, were calculated for all demographic and clinical variables, as well as for items assessing diagnosis experience, emotional responses, and family impact.

For inferential analysis, associations between categorical variables were examined using Pearson’s chi-square tests. Variables selected for correlation analyses were those identified *a priori* as potentially relevant based on the literature review and theoretical framework, including: provision of information at diagnosis (excellent explanation vs. no explanation), maternal employment status (working vs. not working), partner’s education level (below high school, high school, bachelor’s degree and higher), and the informant of diagnosis (obstetrician/gynecologist, pediatrician, general physician/nurse, family/friends). These grouping variables were then examined in relation to various maternal emotional and family outcome measures.

Given the exploratory nature of this study and the number of statistical tests performed across multiple emotional and social outcomes, we did not apply a correction for multiple comparisons, such as the Bonferroni correction. This decision was made to avoid inflating Type II error and missing potentially meaningful associations in this understudied population. However, we acknowledge that this approach increases the risk of Type I error, and therefore, findings with *p*-values below 0.05 should be interpreted as hypothesis-generating rather than confirmatory. To provide a more complete picture of the findings, we have included effect sizes (Cramér’s V) for all significant associations, which offer a measure of the strength of association independent of sample size. All *p*-values are reported as two-tailed.

**Figure 1:**
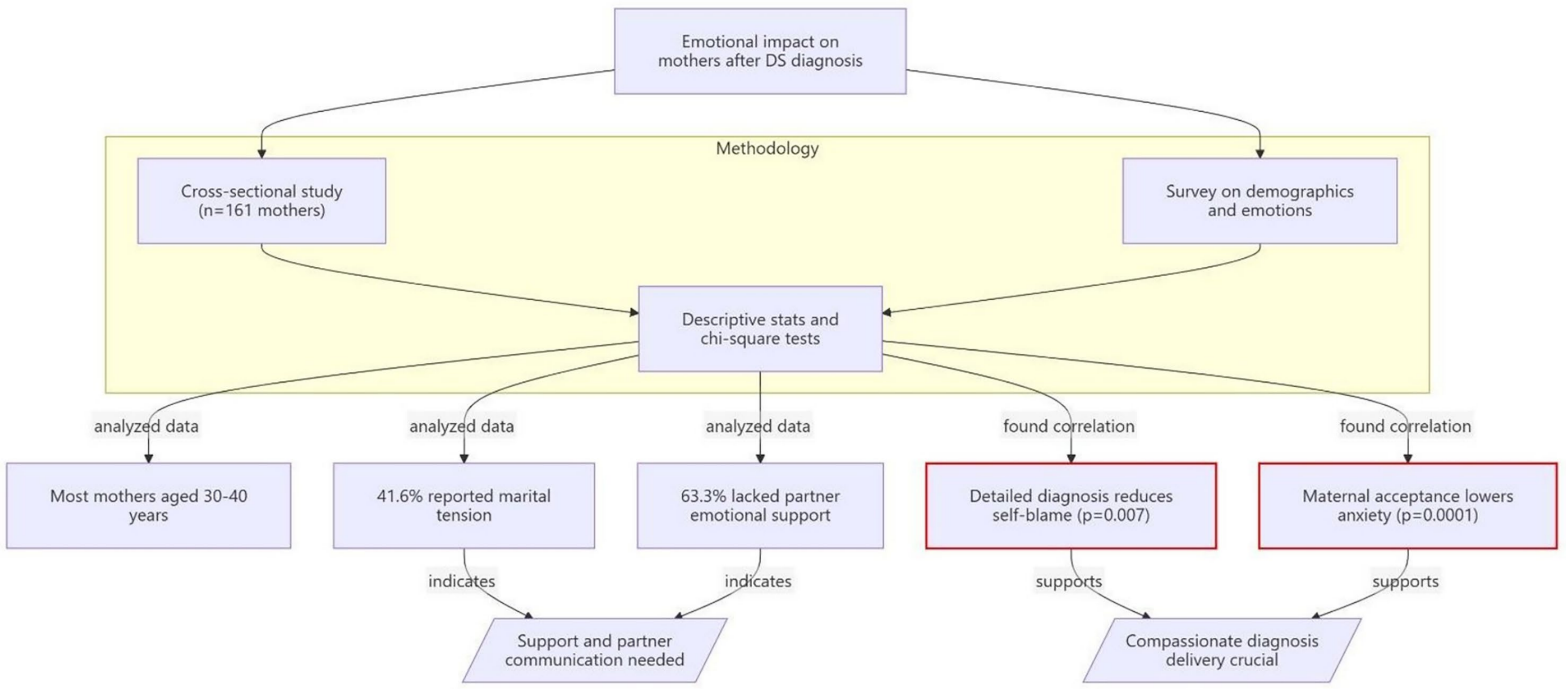
Study design and participant flow. Flow diagram summarizing the cross-sectional design, participant inclusion (*n* = 161 mothers of children with Down syndrome in Saudi Arabia), survey components, and major outcome measures.

A post-hoc power analysis was not conducted, as the sample size was determined by feasibility and response rate rather than by formal power calculation. The final sample of 161 participants exceeds the minimum recommended for exploratory chi-square analyses with anticipated small to medium effect sizes. The overall study design, participant flow, and key outcome measures are summarized in Figure 1.

## Results

### Demographic characteristics

A total of 161 mothers of children with DS completed the survey. The demographic characteristics of the participants and their families are summarized in Table 1. Most of the children were Saudi nationals (80.5%) and male (61.5%). Most children (58.4%) were aged 1–5 years. Mothers were most frequently between 30 and 40 years old (47.5%) at the time of the child’s birth, while their partners were predominantly aged 40–50 years (49.7%). A high level of education was common, with 52.2% of mothers and 44.7% of partners holding a bachelor’s degree. Consanguinity was reported by 29.8% of participants. One-third of the mothers (33.5%) were employed, with most working mothers (35.4%) spending 4–8 h per day outside the home.

### Diagnosis experience and initial emotional impact

**Figure 2:**
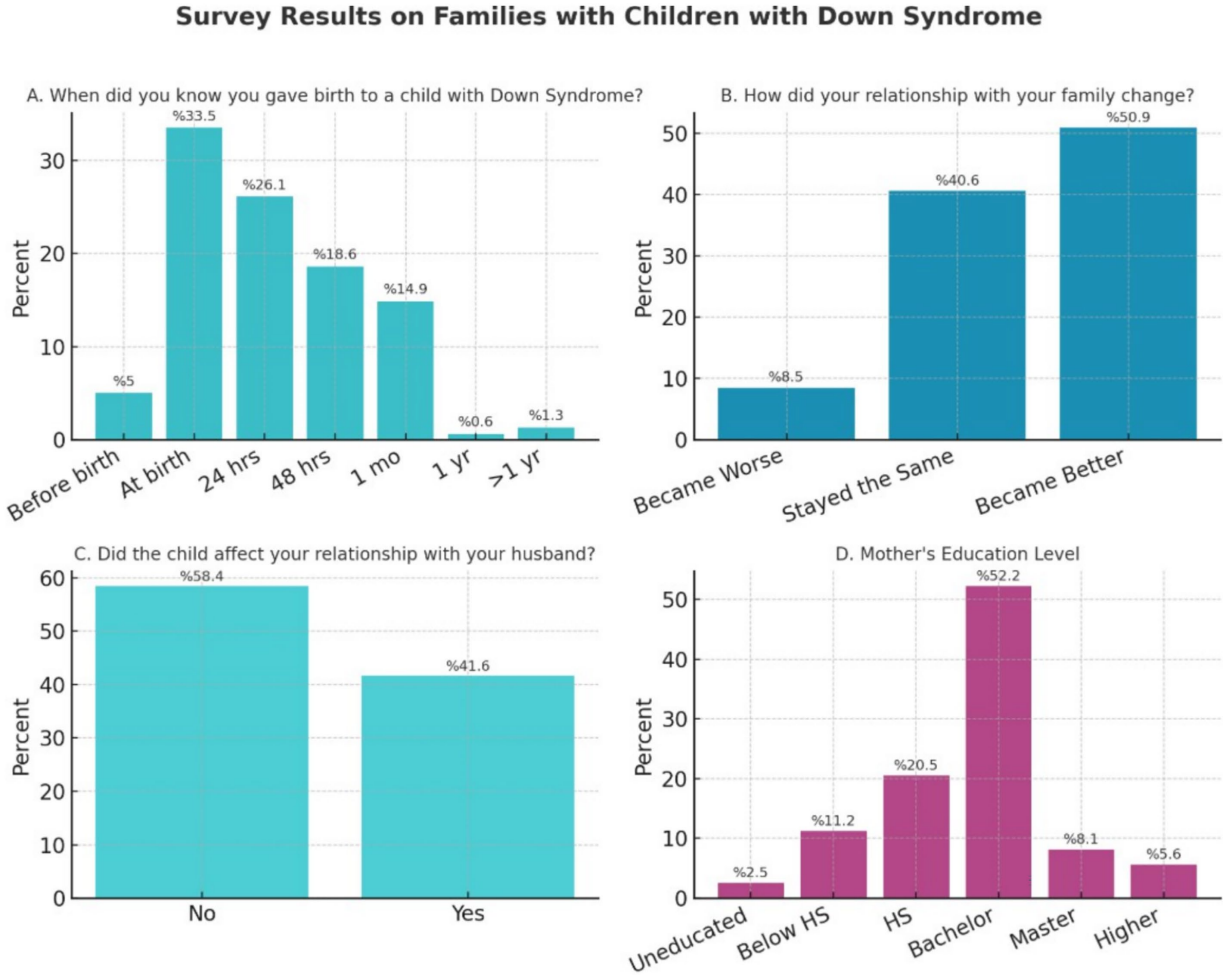
Diagnosis experience and family impact. This figure illustrates the proportion of mothers whose initial negative emotional responses (sadness, shock, and depression) persisted for more than 1 year following diagnosis, grouped by their partner’s educational attainment. Lower partner education was associated with longer duration of maternal distress. **(A)** Circumstances of Down syndrome diagnosis disclosure. **(B)** Impact of diagnosis on family dynamics and social life. **(C)** Partner reactions to the Down syndrome diagnosis. **(D)** Duration of maternal negative feelings in relation to partner’s education level (HS, High School).

The circumstances of the diagnosis disclosure are detailed in Figure 2A. Most mothers received the diagnosis postnatally, with only 5% learning of it prenatally. This low rate of prenatal diagnosis may reflect both the structure of prenatal screening services in Saudi Arabia and cultural considerations around prenatal testing. While prenatal screening is available in major urban centers, access varies geographically, and some families may decline screening due to religious or cultural beliefs (Al-Aqeel, 2016). The predominance of postnatal diagnosis has significant implications for family preparation and support, as parents who receive the diagnosis postnatally must navigate the initial shock of discovery without the opportunity for pre-birth education and planning (Lou et al., 2019). Pediatricians were the most common professionals to deliver the news (62.7%). While 53.4% of mothers reported being informed in a “good, merciful” way, a significant proportion described the delivery as “uncaring/unsympathetic” (26.6%) or “horrible” (19.9%). A critical finding was that 75.8% of mothers received no care guidelines or explanatory resources at the time of diagnosis (Table 2). The initial emotional reactions were profound. Sadness (54.7%) and shock (43%) were the most frequently reported feelings. For 21.1% of mothers, these negative feelings persisted at the time of the survey. In contrast, 57.1% of mothers felt their partner accepted the news more readily upon hearing it.

**Table 2:** Maternal experience of Down syndrome diagnosis disclosure and initial reaction.

Variable	Group	Frequency (n)	Percent (%)
Timing of diagnosis	Before birth	8	5.0
	At birth	54	33.5
	24 h after birth	42	26.1
	48 h after birth	30	18.6
	One month after birth	24	14.9
	More than one year after birth	3	1.9
Professional informing mother	Pediatrician	101	62.7
	Gynecologist/Obsetrician	20	12.4
	General physician and nurse	10	6.2
	Family and friends	30	18.6
Manner of diagnosis delivery	Good, merciful way	86	53.4
	Uncaring/unsympathetic way	43	26.7
	Horrible way	32	19.9
Provision of resources/Care guidelines	No explanation or resources provided	122	75.8
	Excellent explanation about DS	39	24.2
Initial maternal reaction	Sadness	88	54.7
	Shock	70	43.5
	Did not know what DS was	46	28.6
	Acceptance	50	31.1
	Depression	34	21.1

### Ongoing emotional burden and social impact

Despite initial distress, most mothers (88.8%) reported having a “very close” relationship with their child. However, high levels of ongoing psychological burden were evident: 72.7% reported anxiety, 71.4% felt overwhelmed by their responsibilities, 62.1% experienced insomnia, and 55.3% reported anger. Conversely, most mothers did not feel guilt (77.6%) toward their child.

The diagnosis had a significant impact on family dynamics and social life, as illustrated in Figure 2B. Approximately 42 % (41.6%) of mothers reported that having a child with DS affected their marital relationship, and of those, 58.3% stated the relationship had weakened. A majority (63.3%) reported a lack of emotional support from their partners. Socially, 45.3% reported an affected social life, though relationships with the mother’s own family improved for 50.9% of participants.

Partner reactions to the diagnosis, a key factor in family adaptation, are further broken down by the informant in Figure 2C.

### The impact of information and supportive communication

Correlation analyses revealed that the quality of information and support provided at diagnosis was significantly associated with maternal well-being. Receiving an excellent explanation about DS was associated with several positive outcomes, including reduced fear of societal perception (*p* = 0.045, *φ* = 0.158), lower levels of guilt (*p* = 0.003, *φ* = 0.233), less persistent anxiety (*p* = 0.005, *φ* = 0.221), and reduced self-blame (*p* = 0.007, *φ* = 0.212) (Table 3). These effect sizes are small to small-to-moderate according to conventional interpretations (*φ* = 0.10 small, 0.30 medium), suggesting that while the quality of explanation is meaningful, other factors also contribute to these outcomes.

**Table 3:** Association between provision of information at diagnosis and subsequent maternal feelings and behaviors.

Maternal feeling/behavior	Group	Excellent explanation about DS (*n* = 39)	No explanation/resources provided (*n* = 122)	*p*-value
Are you scared of how society will view your child?	No	31 (29.0%)	76 (71.0%)	0.045*
	Yes	8 (14.8%)	46 (85.2%)	
Do you feel guilty towards your child?	No	37 (29.6%)	88 (70.4%)	0.003*
	Yes	2 (5.6%)	34 (94.4%)	
Do you feel nervous/anxious all the time?	No	29 (33.0%)	59 (67.0%)	0.005*
	Yes	10 (13.7%)	63 (86.3%)	
Did you or do you still blame yourself?	No	33 (30.6%)	75 (69.4%)	0.007*
	Yes	6 (11.3%)	47 (88.7%)	

*Indicate statistically significant *p*-value. Effect size φ (phi) is reported for all 2 × 2.

Furthermore, the identity of the informant was significantly associated with partner support; when a physician delivered the news, partners were more likely to calm the mother (*p* = 0.046, φ = 0.157). The manner of delivery was also significantly associated with the informant (*p* = 0.001, Cramér’s V = 0.279), and the provision of resources was dependent on who broke the news (*p* = 0.004, Cramér’s V = 0.253) (Table 4). These effect sizes indicate small-to-moderate associations.

**Table 4:** Association between the informant of the diagnosis and key communication and support outcomes.

Variable	Group	OB/GYN (*n* = 20)	Pediatrician (*n* = 101)	General physician and nurse (*n* = 10)	Family and friends (*n* = 30)	*p*-value
How was the way to tell you?	Good, Merciful Way	16 (18.6%)	40 (46.5%)	5 (5.8%)	25 (29.1%)	0.001*
	Uncaring/Unsympathetic Way	2 (4.7%)	35 (81.4%)	4 (9.3%)	2 (4.7%)	
	Horrible Way	2 (6.3%)	26 (81.3%)	1 (3.1%)	3 (9.4%)	
When the news broke, what resources were provided?	Excellent explanation	10 (25.6%)	19 (48.7%)	0 (0.0%)	10 (25.6%)	0.004*
	No explanation/resources	10 (8.2%)	82 (67.2%)	10 (8.2%)	20 (16.4%)	
What was your husband’s reaction? (Calmed you down)	No	16 (16.2%)	65 (65.7%)	4 (4.0%)	14 (14.1%)	0.046*
	Yes	4 (6.5%)	36 (58.1%)	6 (9.7%)	16 (25.8%)	

*Indicate statistically significant *p*-value.

### The dual burden of employment

Maternal employment status was a significant factor associated with heightened distress (Table 5). Being a working mother was strongly correlated with a more negatively affected relationship with their partner (*p* = 0.004), higher rates of depression (*p* = 0.0001), sadness (*p* = 0.030), guilt (*p* = 0.018), anger (*p* = 0.039), and self-blame (*p* = 0.010). Furthermore, working mothers were significantly more likely to report feeling embarrassed (*p* = 0.043) and regretting having a child with DS (*p* = 0.014) (Table 6). As expected, a large majority (62.5%) of working mothers reported that their child affected their work life, primarily through losing focus (71.4% of those affected) (*p* = 0.0001).

**Table 5:** Associations between maternal employment status and psychosocial outcomes.

Variable	Group	Non-working mother (*n* = 107)	Working mother (*n* = 54)	*p*-value	Cramér’s V
Relationship and partner support					
Did the child affect your relationship with your husband?	No	71 (75.5%)	23 (24.5%)	0.004*	0.227
	Yes	36 (53.7%)	31 (46.3%)		
What was your husband’s reaction? (Acceptance)	No	38 (55.1%)	31 (44.9%)	0.008*	0.210
	Yes	69 (75.0%)	23 (25.0%)		
Maternal emotional well-being					
How did you feel? (Depressed)	No	93 (73.2%)	34 (26.8%)	0.0001*	0.294
	Yes	14 (41.2%)	20 (58.8%)		
How did you feel? (Sadness)	No	55 (75.3%)	18 (24.7%)	0.030*	0.171
	Yes	52 (59.1%)	36 (40.9%)		
Do you feel guilty?	No	89 (71.2%)	36 (28.8%)	0.018*	0.187
	Yes	18 (50.0%)	18 (50.0%)		
Did you suffer from anger?	No	54 (75.0%)	18 (25.0%)	0.039*	0.163
	Yes	53 (59.6%)	36 (40.4%)		
Did you blame yourself?	No	79 (73.1%)	29 (26.9%)	0.010*	0.202
	Yes	28 (52.8%)	25 (47.2%)		
Impact on career					
Did your child affect your work life?	No	89 (78.8%)	24 (21.2%)	0.0001*	0.381
	Yes	18 (37.5%)	30 (62.5%)		
Did you lose focus at your job?	No	17 (54.8%)	14 (45.2%)	0.041*	0.304
	Yes	8 (28.6%)	20 (71.4%)		

Appendix: *Indicates statistically significant at *p* < 0.05. Cramér’s V effect size interpretation: 0.10–0.30 = small effect, 0.30–0.50 = medium effect, >0.50 = large effect.

**Table 6:** Association between maternal employment status and feelings towards the child with Down syndrome.

Variable	Group	Non-working mother (*n* = 107)	Working mother (*n* = 54)	*p*-value
I am embarrassed of my child with Down syndrome	Disagree	77 (61.6%)	48 (38.4%)	0.043*
	Neutral	20 (87.0%)	3 (13.0%)	
	Agree	10 (76.9%)	3 (23.1%)	
I regret having a child with Down syndrome	Disagree	71 (64.0%)	40 (36.0%)	0.014*
	Neutral	24 (88.9%)	3 (11.1%)	
	Agree	12 (52.2%)	11 (47.8%)	

*Indicate statistically significant *p*-value.

### The role of partner education and long-term adaptation

The partner’s education level showed specific, significant correlations. Mothers were more likely to be employed if their partner held a bachelor’s degree or higher (*p* = 0.032, Cramér’s V = 0.205). Furthermore, higher partner education was significantly associated with the mother’s work life being affected by the child’s needs (*p* = 0.015, Cramér’s V = 0.227) (Table 7). Both associations represent small effect sizes.

**Table 7:** Association between partner’s education level and maternal employment outcomes.

Variable	Group	Below high school (*n* = 20)	High school (*n* = 33)	Bachelor’s degree and higher (*n* = 108)	*p*-value
Are you a working mother?	No	17 (15.9%)	25 (23.4%)	65 (60.7%)	0.032*
	Yes	3 (5.6%)	8 (14.8%)	43 (79.6%)	
Did your child affect your work life?	No	17 (15.0%)	26 (23.0%)	70 (61.9%)	0.015*
	Yes	3 (6.3%)	7 (14.6%)	38 (79.2%)	

*Indicate statistically significant *p*-value.

Regarding the duration of maternal negative feelings, as illustrated in Figure 2D, mothers whose partners held lower educational attainment (below high school or high school only) more frequently reported that their negative feelings persisted for longer durations (1 year or more) following the diagnosis. In contrast, mothers with partners holding bachelor’s degrees or higher more often reported that negative feelings resolved within the first year. Similarly, partner non-acceptance of the diagnosis was reported more frequently and for longer durations among families where partners had lower educational attainment. These patterns suggest that socioeconomic resources, as reflected in partner education, may play a role in the family’s adaptation trajectory, though the cross-sectional nature of these data means that these associations should be interpreted descriptively rather than causally. No other significant correlations were found between partner education and emotional or relational variables.

## Discussion

**Figure 3:**
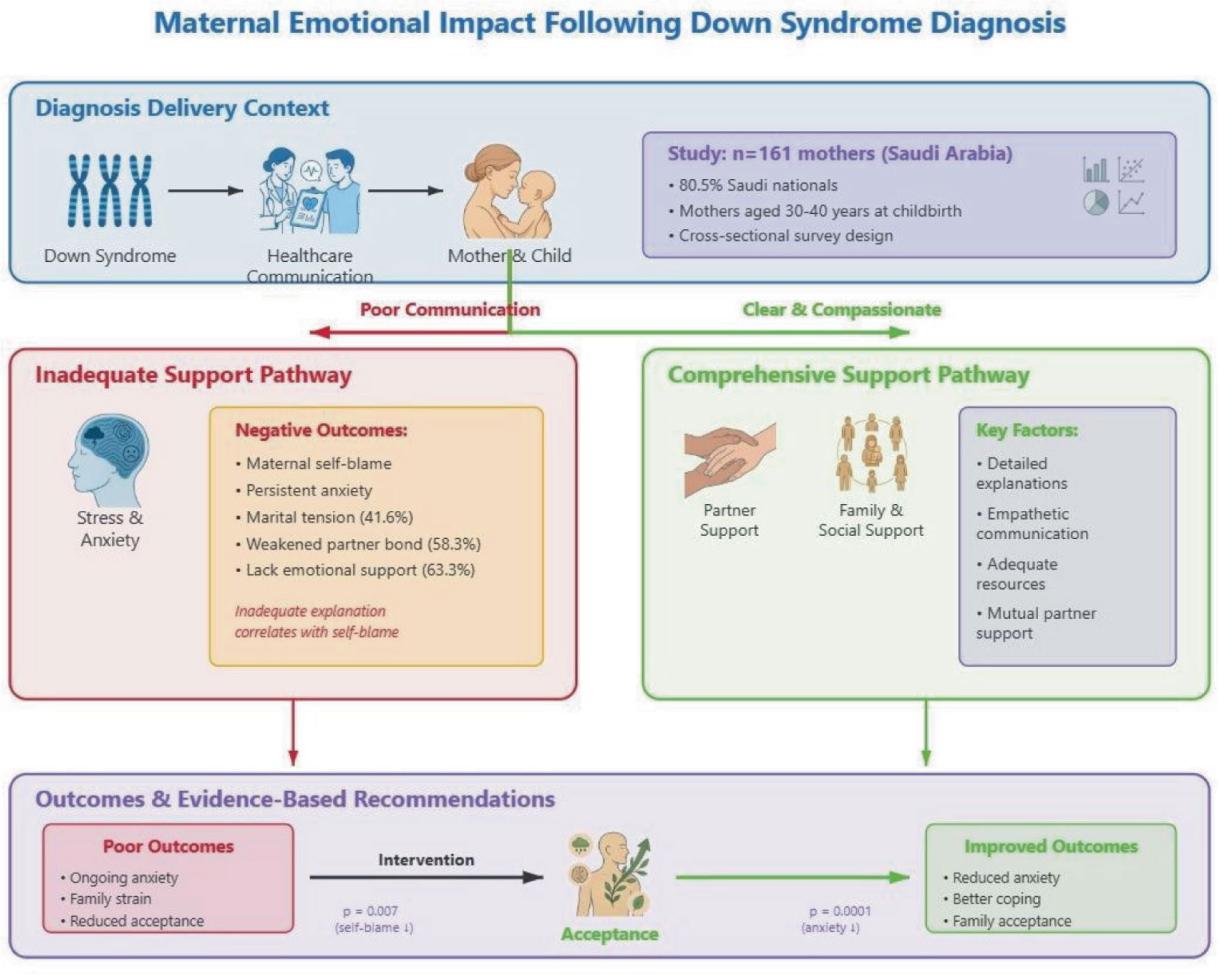
Conceptual framework of maternal emotional impact following Down syndrome diagnosis. This figure illustrates one potential interpretation of the relationships examined in this cross-sectional study. Solid arrows represent associations identified in our analyses; dashed arrows indicate hypothesized pathways suggested by the literature that require longitudinal research to substantiate. Given the correlational nature of this study, causal relationships cannot be inferred, and this framework should be viewed as a guide for future hypothesis-driven investigation.

The literature indicates that caregivers of children with DS experience considerable stress associated with the diagnosis and related comorbidities, and research has identified associations between caregiver well-being and children’s developmental outcomes across multiple domains (Mbugua et al., 2011) (Figure 3). In addition, it has been shown that caring for a child with DS harms the caregiver’s physical and mental well-being, everyday life, connections with family and friends, career, social life, leisure activities, and financial stability (Mbugua et al., 2011; AlShatti et al., 2021). These findings are supported by those of other studies, which have demonstrated that coping with health issues and related behavioral, motor, cognitive, and developmental delays of children with DS is emotionally taxing for their caregivers (Barros et al., 2017).

The high proportion of mothers reporting inadequate information at diagnosis, coupled with the substantial number who perceived communication as unsympathetic, points to systemic gaps in how diagnostic news is delivered and supported. Similar findings have been documented across diverse cultural contexts, suggesting that inadequate diagnosis disclosure practices represent a global challenge in pediatric care (Skotko et al., 2009; Grane et al., 2023). That over 70 % of participants reported feeling overwhelmed is perhaps unsurprising given these early experiences and aligns with research demonstrating that the quality of initial communication shapes subsequent parental adjustment (Huiracocha et al., 2017; Skotko, 2005). These findings suggest that the clinical encounter at diagnosis represents not merely a moment of information transfer but a foundational experience with potential long-term implications for family coping. While the need for comprehensive information and guidance for families of affected individuals is well established in the literature (Kalyoncu et al., 2018), our results suggest that such informational and emotional support is not consistently provided in this context. Research has demonstrated that physician empathy strengthens therapeutic relationships and fosters patient trust (Lussier and Richard, 2010), yet our findings indicate that there may be barriers, whether related to training, time constraints, or systemic protocols, that prevent this ideal from being realized consistently. The high proportion of participants reporting feeling overwhelmed (71.4%) may reflect, at least in part, the absence of adequate informational and emotional support at the time of diagnosis, as well as limited discussion of available caregiver resources. Moreover, among the participants, 27.3% shared that there was an absence of support provided, and such support promotes positive mental health (Marshall et al., 2015). Furthermore, more than half of the participants (63.3%) reported that they received no support from their partner. However, studies have indicated that partner support can play a significant role in promoting health, facilitating positive behaviors, and changing maladaptive health beliefs (Mitchell et al., 2015). Our findings echo those of such studies, which have emphasized the importance of having a supportive partner.

Some of the participants indicated that their career had been affected (29.8%), with 47.5% indicating that they lost focus at work. Some had to implement changes in their work life, such as decreasing their work hours (48.3%) or quitting their job (31.9%). These findings are comparable to those reported by Schieve et al. (2011), who found that in 40% of families, a family member quit their job due to the needs of their child with DS. Working parents face additional challenges; nearly half of the working participants in this study reported that their relationship was negatively affected, which led to 58.8% developing depression (Schieve et al., 2011).

The diagnosis of DS is associated with challenges that affect multiple aspects of family life, including the general health of family members. Notably, our findings suggest that some of these challenges may be less frequently reported when families receive comprehensive information at the time of diagnosis, though further research would be needed to establish whether this relationship is causal. In this study, nearly one-third of the participants reported feeling less scared when enough information was provided. This echoes the previous recommendation for physicians to actively engage with the family. A further 33% of the participants reported feeling less stressed and guilty when adequate information was provided. Strikingly, 88.7% of those who did not receive adequate information blamed themselves, and 91% of their partners developed depression.

The participants’ responses in this cross-sectional study also revealed that they experienced negative emotions when they were informed about their child’s diagnosis and that they potentially required additional psychiatric and emotional support to help them cope. Nearly half of our participants (57.1%) reported that their partner accepted the news better than themselves, and our data revealed that in 63.3% of the couples, the partners were not providing emotional support to each other. Given the importance of partner-based support, which involves one partner providing support and the other receiving support, such support should be encouraged, and supportive measures should be provided to the more affected partner (Button et al., 2001).

When we analyzed the correlations between different variables, we continued to observe that the participants and their partners demonstrated negative feelings, including embarrassment, guilt, sadness, anger, and self-blame. Others have suggested that such emotions have an indirect effect on the child’s emotions through permissive parenting, especially parental stress (Faught et al., 2022). The consistent pattern of associations between maternal employment and multiple indicators of psychological distress warrants careful consideration. While employment might be expected to serve as a source of fulfillment and respite, our findings suggest that for many mothers in this context, paid work outside the home may introduce additional strain rather than relief. This could reflect the practical challenges of balancing caregiving with professional responsibilities, particularly in the absence of adequate workplace accommodations or flexible arrangements. It may also point to unmeasured factors, such as the possibility that working mothers face greater overall demands or have partners who are less involved in caregiving. These findings underscore the need for workplace policies that better support employed mothers of children with disabilities, including flexible hours, remote work options, and access to reliable respite care.

The role of physician communication also emerged as significantly associated with maternal adjustment. Mothers who perceived their physician as competent and compassionate in sharing the diagnosis reported lower levels of guilt, self-blame, and persistent anxiety. This aligns with a growing body of literature emphasizing that how diagnostic news is delivered can be as important as the news itself (Fonseca et al., 2014; Van Riper and Choi, 2011). Our findings extend this literature by quantifying the strength of these associations (with effect sizes ranging from small to moderate) and demonstrating that the impact of communication quality extends beyond immediate emotional responses to influence longer-term adjustment patterns. These findings suggest the value of integrating structured communication training into medical education and ensuring that pediatricians, genetic counselors, and social workers have access to evidence-based guidelines for breaking bad news sensitively and connecting families with appropriate support services. Interventional studies would be valuable to establish whether such training translates into measurable improvements in parental outcomes. Our results also indicate that delivering adequate information to parents of children with DS is vital; it helps them feel less scared about how society will view their child. Thus, we recommend creating an information pack that pediatricians can provide to parents of children with DS that details how to manage their child’s needs, including their medical, physiological, and psychological needs.

The consistent associations observed between socioeconomic indicators, particularly partner education, and maternal outcomes warrant further exploration. While higher partner education was associated with maternal employment and with the mother’s work life being affected by the child’s needs, no direct associations were found between partner education and maternal emotional outcomes. This suggests that socioeconomic resources may influence family adaptation primarily through practical pathways, such as enabling access to services or flexible work arrangements, rather than directly modifying emotional responses. These nuanced findings underscore the need for future research to examine the mechanisms through which socioeconomic factors shape family adaptation (Emerson et al., 2006).

The finding that only 5% of mothers received a prenatal diagnosis warrants attention in light of Saudi Arabia’s evolving prenatal screening policies. International guidelines recommend offering prenatal screening for DS to all pregnant women, with invasive diagnostic testing available following positive screens (American College of Obstetricians and Gynecologists’ Committee on Practice Bulletins—Obstetrics, Committee on Genetics, Society for Maternal-Fetal Medicine, 2020). However, implementation varies across healthcare settings, and cultural factors may influence both provider recommendations and patient decisions. The predominance of postnatal diagnosis in our sample suggests that opportunities for pre-birth parental education, preparation, and informed decision-making are being missed. Future research should examine barriers to prenatal screening access in Saudi Arabia and explore how pre-birth diagnosis, when appropriately supported, might facilitate more positive family adaptation.

Beyond partner support, our findings reveal multiple sources of stress that warrant consideration. The high proportion of mothers reporting anger (55.3%), anxiety (72.7%), and feeling overwhelmed (71.4%) suggests that the burden of caregiving extends across multiple domains of life. While we identified a lack of partner support as a significant stressor, other systemic factors likely contribute. Mothers in our sample described challenges within healthcare settings, including unsympathetic communication and inadequate information, that compound the stress of diagnosis. Additionally, while not directly measured, the broader societal context may represent an ongoing source of stress. Previous research in Middle Eastern contexts has documented that parents of children with disabilities face stigmatizing attitudes, limited inclusive educational opportunities, and inadequate workplace accommodations (Abed et al., 2024; Almasri et al., 2018). The finding that 45.3% of mothers reported an affected social life, while over half reported improved relationships with their own families, suggests complex and sometimes contradictory social dynamics. Future research should systematically examine how stressors within healthcare, education, and community settings collectively shape family experiences.

The findings of this study must be understood within the broader context of Saudi Arabia’s evolving healthcare and social service landscape. The Kingdom has made significant strides in recent years through initiatives such as Vision 2030, which includes commitments to strengthening healthcare infrastructure and expanding support services for individuals with disabilities (Saudi vision 2030, 2026). The Rights of Persons with Disabilities Law (Royal Decree No. M/37, 2000) and its subsequent amendments establish legal frameworks for access to healthcare, education, and employment for individuals with disabilities (Abobaker, 2024). However, implementation gaps between policy and practice remain, particularly in the training of healthcare professionals in sensitive diagnosis disclosure and in the availability of coordinated support services across the health, education, and social sectors. Our findings suggest that despite these policy commitments, families continue to experience significant gaps in informational and emotional support at the critical moment of diagnosis.

It is not surprising that most parental emotion-related behaviors (e.g., reactions and expressivity) impact child outcomes. There are, however, many socializing forces besides parents, including siblings, peers, and physicians. Here, we have comprehensively studied the emotional impact of having a child with DS on parents; however, further research is required in this field. An important direction for future research would be to directly examine how specific cultural beliefs and practices, such as religious coping mechanisms, involvement of extended family networks, and community attitudes toward disability, influence maternal adjustment and family adaptation in the Saudi context. While the literature suggests these factors may be significant (Lillard, 1998), they were not empirically measured in the present study and therefore remain important questions for subsequent investigation. The findings of this study have implications for future educational programs and other early intervention programs designed for families of children with DS and other conditions.

Several limitations should be considered when interpreting our findings. First, the cross-sectional design precludes causal inference, and associations identified should be viewed as exploratory rather than confirmatory. Relatedly, the large number of statistical tests performed increases the risk of Type I error; we did not apply corrections for multiple comparisons to prioritize sensitivity in this under-researched population, but significant findings require confirmation in future hypothesis-driven studies. Effect sizes are reported to aid interpretation, with small to medium effects predominating. Second, our data reflect maternal perceptions and recollections of events, which may be influenced by emotional state and the passage of time. These perceptions, while valuable, do not constitute objective measures of physician behavior or healthcare quality. Physicians may operate within systemic constraints, including time pressures, limited training in breaking bad news, or the absence of clear protocols, which were not captured in our study. Third, although cultural and religious factors in Saudi Arabia provided the rationale and interpretive framework for this work, these constructs were not directly measured. Our discussion of extended family support, religious coping, and cultural attitudes toward disability is therefore drawn from the broader literature rather than empirical data from this sample. Future studies incorporating validated measures of these factors would allow for direct testing of their role in maternal adjustment. Fourth, the questionnaire used in this study was developed specifically for this research and did not undergo formal psychometric validation. While we followed a systematic development process including literature review, expert review, and pilot testing, the absence of established reliability and validity measures means that findings should be interpreted with appropriate caution. Finally, the online recruitment strategy may have introduced sampling bias, potentially over-representing mothers with internet access and connections to support networks. The generalizability of our findings to the wider population of mothers of children with DS in Saudi Arabia thus remains to be established.

## Conclusion

In conclusion, this study elucidates the complex interplay between healthcare experiences, familial support, and socioeconomic factors in shaping the emotional well-being of mothers of children with DS in Saudi Arabia. Our findings move beyond merely documenting distress to identify critical, modifiable levers for intervention. The data indicate that the initial clinical encounter represents a significant moment in the parental journey. The manner of diagnosis delivery and the provision of comprehensive information were found to be significantly associated with long-term maternal adjustment, including reported levels of guilt, self-blame, and stress. This underscores an urgent need to replace ad-hoc communication with standardized, empathetic protocols for all healthcare professionals, a recommendation consistent with previous research emphasizing the lasting impact of diagnosis disclosure on family outcomes (Huiracocha et al., 2017; Skotko, 2005; Baird et al., 2000).

Furthermore, the study highlights the familial context of adaptation. The stress reported by mothers, particularly those balancing professional and caregiving responsibilities, frequently co-occurs with limited partner support, suggesting that distress within the couple relationship may be mutually reinforcing. Therefore, effective support must extend beyond the individual mother to encompass the parental dyad, fostering mutual understanding and shared responsibility. Research has demonstrated that partner support plays a crucial role in promoting positive health behaviors and facilitating psychological adjustment (Mitchell et al., 2015; Button et al., 2001), reinforcing the importance of couple-based interventions.

To this end, we recommend a multi-faceted approach drawing on both our findings and the broader literature. First, the implementation of mandatory clinical guidelines for compassionate diagnosis disclosure, as studies have shown that structured protocols improve parental satisfaction and reduce long-term psychological distress (Huiracocha et al., 2017; Kalyoncu et al., 2018). Second, the development and distribution of structured information packs detailing medical, developmental, and support resources, which research indicates can reduce parental anxiety and increase feelings of preparedness (Skotko, 2005; Marshall et al., 2015). Third, the creation of targeted support programs that strengthen couple-based coping and address the unique challenges faced by working mothers, building on evidence that workplace accommodations and partner engagement interventions yield measurable benefits for family well-being (Mitchell et al., 2015; Schieve et al., 2011; Button et al., 2001).

By implementing these measures, healthcare systems and policymakers can significantly mitigate the initial trauma of a DS diagnosis and empower families to build resilient, supportive, and accepting environments for their children.

## Data Availability

The raw data supporting the conclusions of this article will be made available by the authors, without undue reservation.

## References

[ref1] Abed M. G. Abed L. G. Shackelford T. K. (2024). Attitudes towards and communications with people with disabilities in Saudi Arabia: towards the sustainability of a healthy citizenry. Sustainability 16:10061. doi: 10.3390/su162210061

[ref2] Abobaker M. (2024). Developing Saudi Arabia’s disability rights legislation in compliance with the United Nations convention on the rights of persons with disabilities in education system. J. Infrastruct. Policy Dev. 8:9343. doi: 10.24294/jipd9343

[ref3] Al-Aqeel A. I. (2016). Ethical considerations in prenatal diagnosis in Saudi Arabia. J. Saudi Heart Assoc. 28, 192–197.

[ref4] Al-Gamal E. Long T. (2013). Psychological distress and perceived support among Jordanian parents living with a child with cerebral palsy: a cross-sectional study. Scand. J. Caring Sci. 27, 624–631. doi: 10.1111/j.1471-6712.2012.01071.x 22924549

[ref5] Al-Jadid M. S. Robert A. A. (2010). Determinants of length of stay in an inpatient stroke rehabilitation unit in Saudi Arabia. Saudi Med. J. 31, 189–192. 20174737

[ref6] Almalki S. (2020). “Parenting practices in Saudi Arabia: gender-role modeling,” in Parents and Caregivers Across Cultures, eds. Ashdown B. K. Faherty A. N. (Cham: Springer International Publishing), 231–246.

[ref7] Almasri N. A. An M. Palisano R. J. (2018). Parents' perception of receiving family-centered care for their children with physical disabilities: a meta-analysis. Phys. Occup. Ther. Pediatr. 38, 427–443. doi: 10.1080/01942638.2017.1337664, 28753054

[ref8] Alqarawi N. Alhamidi S. A. Alsadoun A. Alasqah I. Mahmud I. (2023). Challenges of having a child with congenital anomalies in Saudi Arabia: a qualitative exploration of mothers' experience. Front. Public Health 11:1111171. doi: 10.3389/fpubh.2023.1111171, 37168071 PMC10166135

[ref9] AlShatti A. AlKandari D. AlMutairi H. AlEbrahim D. AlMutairi A. AlAnsari D. . (2021). Caregivers' perceptions and experience of caring for persons with down syndrome in Kuwait: a qualitative study. Int. J. Dev. Disabil. 67, 381–390. doi: 10.1080/20473869.2021.1910780, 34570835 PMC8451671

[ref10] American College of Obstetricians and Gynecologists’ Committee on Practice Bulletins—Obstetrics, Committee on Genetics, Society for Maternal-Fetal Medicine (2020). Screening for fetal chromosomal abnormalities: ACOG practice bulletin, number 226. Obstet. Gynecol. 136, e48–e69. doi: 10.1097/AOG.0000000000004084, 32804883

[ref11] Baird G. McConachie H. Scrutton D. (2000). Parents' perceptions of disclosure of the diagnosis of cerebral palsy. Arch. Dis. Child. 83, 475–480. doi: 10.1136/adc.83.6.475, 11087279 PMC1718571

[ref12] Barros A. L. O. Barros A. O. Barros G. L. M. Santos M. T. B. R. (2017). Burden of caregivers of children and adolescents with down syndrome. Ciênc. Saúde Colet. 22, 3625–3634. doi: 10.1590/1413-812320172211.31102016, 29211168

[ref13] Button S. Pianta R. C. Marvin R. S. (2001). Partner support and maternal stress in families raising young children with cerebral palsy. J. Dev. Phys. Disabil. 13, 61–81. doi: 10.1023/a:1026509400487

[ref14] Cuskelly M. Hauser-Cram P. Van Riper M. (2009). Families of children with down syndrome: what we know and what we need to know. Down Syndr. Res. Pract. 12, 105–116.

[ref15] Emerson E. Hatton C. Llewellyn G. Blacher J. Graham H. (2006). Socio-economic position, household composition, health status and indicators of the well-being of mothers of children with and without intellectual disabilities. J. Intellect. Disabil. Res. 50, 862–873. doi: 10.1111/j.1365-2788.2006.00900.x 17100947

[ref16] Faught G. G. Phillips B. A. Conners F. A. (2022). Permissive parenting mediates parental stress and child emotions in families of children with down syndrome. J. Appl. Res. Intellect. Disabil. 35, 1418–1428. doi: 10.1111/jar.13031, 36054429 PMC10017012

[ref17] Fonseca A. Nazaré B. Canavarro M. C. (2014). Parenting an infant with a congenital anomaly: an exploratory study on patterns of adjustment from diagnosis to six months post birth. J. Child Health Care 18, 111–122. doi: 10.1177/1367493512473856, 23728929

[ref18] Fortier L. M. Wanlass R. L. (1984). Family crisis following the diagnosis of a handicapped child. Fam. Relat. 33, 13–24. doi: 10.2307/584585

[ref19] Grane F. M. Lynn F. Balfe J. Molloy E. Marsh L. (2023). Down syndrome: parental experiences of a postnatal diagnosis. J. Intellect. Disabil. 27, 1032–1044. doi: 10.1177/17446295221106151, 35698902 PMC10647884

[ref20] Huiracocha L. Almeida C. Huiracocha K. Arteaga J. Arteaga A. Blume S. (2017). Parenting children with down syndrome: societal influences. J. Child Health Care 21, 488–497. doi: 10.1177/1367493517727131, 29110530 PMC5697561

[ref21] Kalyoncu I. O. Giray F. E. Tanboga I. (2018). Parent's attitudes and knowledge on oral health in a group of individual with down syndrome in Turkey. J. Pak. Med. Assoc. 68, 1368–1372. 30317267

[ref22] Lazarus R. S. Folkman S. (1984). Stress, Appraisal, and Coping. New York: Springer publishing company.

[ref23] lden E. (1997). Reviews: Robert J. Thompson, Jr. & Kathryn E. Gustafson, adaptation to chronic childhood illness Washington, DC: American Psychological Association, 1996, 382 pp. US$49.95 (hbk); ISBN 1-55798-327-5. J. Health Psychol. 2, 265–266. doi: 10.1177/135910539700200222

[ref24] Lillard A. (1998). Ethnopsychologies: cultural variations in theories of mind. Psychol. Bull. 123, 3–32. doi: 10.1037/0033-2909.123.1.3, 9461850

[ref25] Lou S. Carstensen K. Vogel I. Hvidman L. Nielsen C. P. Lanther M. . (2019). Receiving a prenatal diagnosis of down syndrome by phone: a qualitative study of the experiences of pregnant couples. BMJ Open 9:e026825. doi: 10.1136/bmjopen-2018-026825, 30867204 PMC6429881

[ref26] Lussier M. T. Richard C. (2010). Should family physicians be empathetic? YES. Can. Fam. Physician 56, 740–e289. 20705874 PMC2920767

[ref27] Marshall J. Tanner J. P. Kozyr Y. A. Kirby R. S. (2015). Services and supports for young children with down syndrome: parent and provider perspectives. Child Care Health Dev. 41, 365–373. doi: 10.1111/cch.12162, 24912377

[ref28] Mbugua M. N. Kuria M. W. Ndetei D. M. (2011). The prevalence of depression among family caregivers of children with intellectual disability in a rural setting in Kenya. Int. J. Fam. Med. 2011:534513. doi: 10.1155/2011/534513, 22295186 PMC3263839

[ref29] McCubbin H. I. McCubbin M. A. Thompson A. I. Han S. Y. Allen C. T. (1997). Families under stress: what makes them resilient. J. Fam. Consum. Sci. 89, 2–11.

[ref30] Mitchell D. B. Hauser-Cram P. Crossman M. K. (2015). Relationship dimensions of the 'down syndrome advantage'. J. Intellect. Disabil. Res. 59, 506–518. doi: 10.1111/jir.12153, 25070618 PMC4309742

[ref31] Mulcahy H. Savage E. (2016). Uncertainty: a little bit not sure. Parental concern about child growth or development. J. Child Health Care 20, 333–343. doi: 10.1177/1367493515587059 26105061

[ref32] Saudi vision 2030 (2026). Saudi vision 2030 - health sector transformation program. Available online at: https://www.vision2030.gov.sa/en/explore/programs/health-sector-transformation-program (Accessed March 20, 2026).

[ref33] Schieve L. A. Boulet S. L. Kogan M. D. Van Naarden-Braun K. Boyle C. A. (2011). A population-based assessment of the health, functional status, and consequent family impact among children with down syndrome. Disabil. Health J. 4, 68–77. doi: 10.1016/j.dhjo.2010.06.001, 21419370

[ref34] Skotko B. (2005). Mothers of children with down syndrome reflect on their postnatal support. Pediatrics 115, 64–77. doi: 10.1542/peds.2004-0928 15629983

[ref35] Skotko B. G. Capone G. T. Kishnani P. S. Down Syndrome Diagnosis Study Group (2009). Postnatal diagnosis of down syndrome: synthesis of the evidence on how best to deliver the news. Pediatrics 124, e751–e758. doi: 10.1542/peds.2009-0480, 19786436

[ref36] Van Riper M. Choi H. (2011). Family-provider interactions surrounding the diagnosis of down syndrome. Genet. Med. 13, 714–716. doi: 10.1097/GIM.0b013e3182209f21, 21673579

[ref37] Von Elm E. Altman D. G. Egger M. Pocock S. J. Gøtzsche P. C. Vandenbroucke J. P. (2007). The strengthening the reporting of observational studies in epidemiology (STROBE) statement: guidelines for reporting observational studies. Lancet 370, 1453–1457. doi: 10.1016/s0140-6736(07)61602-x 18064739

